# Coronary flow reserve in patients with left anterior descending artery-left internal mammary artery long patch plasty anastomosis: a prospective study

**DOI:** 10.1186/s13019-015-0247-4

**Published:** 2015-04-02

**Authors:** Ismail Haberal, Onur Gurer, Deniz Ozsoy, Esra Erturk

**Affiliations:** 1 Department of Cardiovascular Surgery, Istanbul University Institute of Cardiology, Istanbul, Turkey; 2 Department of Cardiovascular Surgery, Hospitalium Hospitals Camlıca, Istanbul, Turkey; 3 Department of Cardiovascular Surgery, Mersin Government Hospital, Mersin, Turkey

**Keywords:** Patchplasty, Coronary flow reserve, Transthoracic Doppler echocardiography

## Abstract

**Background:**

We aimed at assessing the efficacy of the patch plasty technique without endarterectomy in patients with diffuse coronary artery. Long anastomosis of the left internal mammary artery graft (LIMA) to the left anterior descending (LAD) artery was performed and examined using transthoracic Doppler echocardiography to detect coronary flow reserve (CFR) and epicardial stenosis.

**Methods:**

Forty-one patients (6 women; mean age, 58 ± 9 years) who underwent coronary artery bypass surgery using the patch plasty technique without endarterectomy were included in the study. Presence of CFR was examined in each patient by transthoracic Doppler echocardiography.

**Results:**

One of the patients (2.4%) died on the first postoperative day. The remaining patients were divided into 2 groups: those with normal CFR (CFR ≥ 2) (n = 35, 88%) and those with low CFR (CFR < 2) (n = 5, 12.0%). The length of patch plasty (3.6 ± 0.82 cm) in the low CFR group was significantly longer than that in the normal CFR group (2.69 ± 0.75 cm). Coronary angiography was performed for the 3 patients with CFR < 2: Two patients showed normal grafts and anastomoses, but the third patient’s distal LAD-LIMA anastomosis was almost 90% occluded.

**Conclusion:**

We elucidated the reliability of the patch plasty without endarterectomy method and transthoracic Doppler echocardiography for detecting the severity of coronary artery disease.

## Background

Disseminated atherosclerosis is based on diffuse coronary artery disease (CAD): If the coronary artery is diffusely diseased, it is usually impossible to find an appropriate lumen for bypass grafting [1]. In such cases, different alternatives need to be considered [2]. One such alternative is coronary endarterectomy, which provides an appropriate lumen for bypass grafting via surgical removal of progressive atheromatous plaques from a narrow or blocked artery [2]. Left anterior descending artery-left internal mammary artery (LAD-LIMA) long patch plasty anastomosis without endarterectomy is another technique that can ensure blood supply to important septal perforator branches of the LAD, which are obstructed or narrowed by atheromatous plaques.

Coronary flow reserve (CFR) represents the rates of coronary blood flow during resting and maximal hyperemia. CFR, measured using echocardiography, helps evaluate the pathological process of coronary stenosis [3]. In this study, we aimed to evaluate the efficacy of the LAD-LIMA long segmental patch plasty without endarterectomy method for patients with diffuse coronary artery disease.

## Methods

### Patients

In this prospective study, 41 patients (35 men, 6 women; mean age, 58 ± 9 years) who underwent coronary artery bypass grafting (CABG) using patch plasty technique without endarterectomy between May 2010 and December 2013 were included consecutively. Patients with dipyridamole allergy, severe chronic obstructive pulmonary disease, arrhythmia, acute coronary syndrome, and dilated cardiomyopathy were excluded from the study. In case of LAD had a severely calcified plaque we felt that this technique should not be performed. The study protocol was approved by the local ethics committee (Istanbul University Istanbul Faculty of Medicine Ethical Committee), and signed informed consent was obtained before the operation.

### Surgical technique

In all cases, cardiopulmonary bypass was performed using a membrane oxygenator, moderate hemodilution, moderate hypothermia (28–30°C), and antegrade-retrograde hyperkalemic blood cardioplegia. The procedure was used to diffuse long-segment stenotic LAD lesions, which needed to be bypassed. After making a small incision near the most distal plaque on the middle LAD, the arteriotomy was extended proximally and distally towards a less-diseased arterial wall. An internal mammary artery graft was prepared by making a longitudinal incision as per the LAD incision length and sutured by the continuous technique with 7.0 monofilament sutures. We used patch plasty without endarterectomy for long segments of LAD-LIMA anastomosis, and each patient was evaluated in the echocardiography laboratory postoperatively. Radial arterial catheters were used for monitoring preoperative and postoperative arterial pressures.

### Echocardiographic measurements

All examinations were performed by the same physician in the sixth postoperative month; dipyridamole echocardiography using the Vivid 7 dimension device (General Electric Waukesha, WI, USA) was used according to the American Society of Echocardiography guidelines and EchoPAC echocardiography program [4]. Diastolic velocity and/or velocity time integral (VTI) levels ≥ 2.0 after dipyridamole infusion were considered as acceptable CFR. We obtained probe flow-directed Doppler sampling of the LAD artery images of the anterior interventricular sulcus by using high frequency (5–7 mH) convex adult echocardiography. Acoustic window images were captured at the interception of the midclavicular line and third-fourth ribs while the patients were lying in the left lateral decubitis position. In the left ventricular long-axis images, LAD mid-distal flow patterns were assessed sliding to lateral localization by the color Doppler ultrasound. In addition to flow-directed Doppler sampling, peak systolic and diastolic flow velocities and diastolic VTI levels were demonstrated. Dipyridamole (0.56 mg/kg) was infused in 4 min, and all the abovementioned measurements were re-performed. Transthoracic echocardiography and flow-directed Doppler sampling of the LAD were performed in all patients. The presence of myocardial hypertrophy leading to microvascular disease was also examined echocardiographically. End-diastolic posterior wall thickness and end-diastolic interventricular septum thickness > 1 cm as well as diastolic malfunction, revealed by echocardiography, were considered indicative of myocardial hypertrophy.

### Statistical analysis

Statistical analysis was performed using the statistical software program SPSS 13.0 (SPSS Inc., Chicago, IL, USA). The data obtained are presented either as mean ± standard deviation or number with percentages. Independent intergroup comparisons were made using Mann–Whitney-*U* test, and dependent variables such as pre- and post-treatment differences were compared using the Wilcoxon test. Relationships between variates were tested using Pearson’s correlation. All P values < 0.05 were considered statistically significant.

## Results

Of the 41 patients, 5 (12.2%) underwent emergency CABG and 36 (87.8%) underwent elective CABG. The clinical properties of all patients are listed in Table 1. The length of patch plasty was found to be 2.83 ± 0.82 (2–4.5) cm. The average number of vessels bypassed was 3.63 ± 0.66 (1–4), the cross-clamp time was 71.49 ± 16.88 (50–100) min, and the total bypass time was 101.17 ± 21.93 (68–154) min. One of the patients (2.4%) died on postoperative day 1 because of a perioperative myocardial infarction.

**Table 1:** **Patients’ profiles**

Patient	(n = 41)	(%)
Age	45–73 years (mean, 58.56 ± 9.40 years)	
Gender		
Male	35	85.4
Female	6	14.6
Familial CAD history	18	43.9
Hypertension	37	90.2
Diabetes mellitus	18	43.9
Obesity	29	70.7
Dyslipidemia	33	80.5
Smoking	27	65.9
Hyperuricemia	0	0
β-Blocker usage	35	85.4
Statin usage	13	31.7
Stable angina Pectoris	6	14.6
Unstable angina Pectoris	26	63.4
Acute myocardial infarction	9	22
PAD	3	7.3
COPD	13	31.7
NYHA CLASS I	2	4.9
NYHA CLASS II	30	73.2
NYHA CLASS III	9	22
CAG (diseased vessels)		
LMCA	4	9.8
LMCA + 3 vessels	2	4.9
3 vessels	22	53.9
2 vessels	16	39.2
1 vessel	0	0
Microvascular area disease	1	2.45
LVEF (%)		
Low (<30%)	0	0
Intermediate (30–45%)	7	17.1
High (>45%)	34	82.9
Diastolic dysfunction		
Grade 1	8	19.5
Grade 2	33	80.5

CAD: Coronary artery disease, PAD: Periferic arterial disease, COPD: Chronic obstructive pulmonary disease, NYHA: New York Heart Association, LMCA: Left main coronary artery, LVEF: Left ventricular ejection fraction.

### Echocardiographic findings

**Figure 1:**
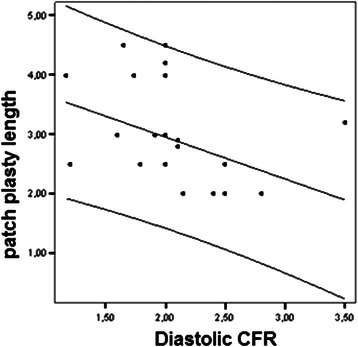
**Correlation between the length (cm) of patch anastomosis (Y axis) and coronary flow reserve (X axis).**

The postoperative left ventricular ejection fraction (EF%) was significantly higher than the preoperative EF% (56.85 ± 6.98 vs 53.83 ± 7.90, P = 0.002)*.* After dipyridamole infusion, the diastolic velocity and/or VTI levels ≥ 2.0 were considered as acceptable CFR. There was a statistically significant reverse correlation between patch plasty length (2.83 ± 0.82 cm) and CFR (2.20 ± 0.47) (P = 0.008) (Figure 1). Patients with grade 2 diastolic dysfunction (n = 33) had a lower CFR than those with grade 1 diastolic dysfunction (n = 8) (1.71 ± 0.36 vs 2.41 ± 0.38, respectively, P = 0.001). CFR of the 5 patients who underwent CABG (n = 40, 12%) were deemed insufficient as per the transthoracic echocardiographic assessments (diastolic CFR < 2; mean, 1.62 ± 0.29).

The patients were categorized into 2 groups according to their CFR: Normal CFR (CFR ≥ 2, n = 35, 88%) and low CFR (CFR < 2, n = 5, 12%). Between the normal and low CFR groups, there were no statistically significant differences between the preoperative and postoperative echocardiographic findings such as left ventricle end-diastolic diameter, left ventricle end-systolic diameter, end-diastolic posterior wall thickness, %EF, and left atrium anteroposterior diameter (Table 2). However, we found a significant correlation between preoperative and postoperative EF% in the normal CFR group (Table 2).

**Table 2:** **Preoperative and postoperative echocardiographic findings and their correlations with coronary flow reserve**

		CFR > 2 Dias n:35	CFR < 2 Dias n:5	P
Left ventricular end diastolic diameter	Preoperative	4.78 ± 0.59	4.46 ± 0.77	0,511
	Postoperative	4.55 ± 0.54	4.6 ± 0.58	0,678
	P	0.151	0.715	
Left ventricular end systolic diameter	Preoperative	3.28 ± 0.51	3.39 ± 0.4	0,742
	Postoperative	3.22 ± 0.53	3.26 ± 0.45	0,901
	P	0.485	0.498	
End diastolic posterior wall thickness	Preoperative	1.52 ± 1.1	1.14 ± 0.14	0.742
	Postoperative	1.09 ± 0.18	1.14 ± 0.15	0.551
	P	0.073	0.999	
% EF by Teicholz method	Preoperative	55.03 ± 6.64	49.2 ± 10.99	0.152
	Postoperative	57.4 ± 6.94	53 ± 6.71	0.188
	P	0.006	0.285	
Left atrium anteroposterior diameter	Preoperative	3.63 ± 0.47	3.67 ± 0.27	0.848
	Postoperative	3.73 ± 0.67	3.8 ± 0.77	0.869
	P	0.211	0.999	

CFR: Coronary flow reserve, EF: Ejection fraction, Dias: Diastolic.

### Coronary angiography

**Figure 2:**
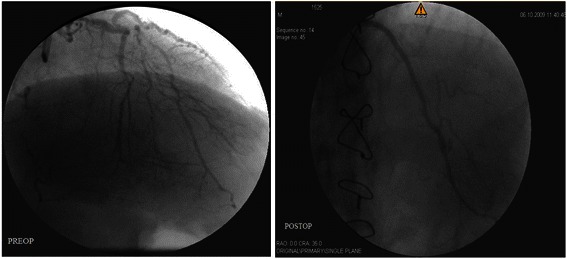
**The preoperative and postoperative coronary angiography images of the left anterior descending artery applied patch plasty technique without endarterectomy.**

According to the results of the echocardiography, CFR of the 5 patients who underwent CABG were insufficient (diastolic CFR < 2; mean, 1.62 ± 0.29). These patients were advised to undergo coronary angiography; however, two of them refused the angiography. Among the remaining 3 patients, the grafts and anastomoses in 2 patients were normal (Figure 2), but the distal LAD-LIMA anastomosis was occluded nearly 90% in the third patient.

## Discussion

Complete revascularization may sometimes not be possible in the diffusely diseased LAD because the conventional bypass grafting techniques to the distal LAD alone cannot provide sufficient blood supply to the side branches, including the diagonal branches and septal perforators. Therefore, techniques such as long coronary arteriotomy, patch reconstruction, or endarterectomy that are performed beyond the diseased segment are necessary to obtain complete revascularization. The frequency of performing coronary endarterectomy differs between centers and is reported to be 2.3%–50% [1]. Although this procedure yields acceptable results, the risk of perioperative events is higher and the long-term prognosis is significantly worse than that of CABG alone [5,6]. Coronary endarterectomy was associated with a higher perioperative mortality (4.4% vs 2.6%) and myocardial infarction (5.4% vs 2.4%) than CABG alone [7]. Coronary reconstruction without endarterectomy is an effective alternative because it does not require removal of the protective endothelium from the coronary artery. We performed LAD-LIMA long patch plasty anastomosis when a simple anastomosis to the distal LAD was not possible because of diffuse lesions. We applied long-segment arteriotomy (2.83 ± 0.82 [2–4.5] cm) to diffusely atherosclerotic LAD vessels and directly anastomosed the LIMA graft to the LAD without endarterectomy, via the long patch plasty anastomosis technique. Thus, it is possible to supply blood to the septal perforating and diagonal arteries. In our study, the occurrence of perioperative myocardial infarction and its associated mortality rate, which was 2.45%, were lower than that of endarterectomy [8-10].

CFR is defined as the ratio of maximal (stimulated) to baseline (resting) coronary blood flow and evaluates the severity of coronary stenosis [11]. Maret et al. [12] showed that a CFR > 2, as noted by transthoracic Doppler echocardiography, in the LAD excluded significant coronary artery disease, which was detected by myocardial perfusion imaging. We proved the reliability of transthoracic Doppler echocardiography for detecting the severity of coronary artery disease. Factors that affect the arterial medial wall thickness and increase the viscosity (thereby affecting microvascular functions), such as arterial hypertension, cardiomyopathy, and diabetes mellitus, may affect the CFR [13,14]. Further, the CFR may decrease without myocardial hypertrophy, as seen in our current patients [15]. In this study, we did not find any statistically significant between a low CFR and left ventricular end-diastolic wall thickness (P = 0.551). In patients with patent grafts, the main cause for low CFR may have been associated with complicated and possibly uncontrolled hypertension.

Despite our important findings, this study has some limitations that need to be acknowledged. Although the left anterior descending artery was viewed using combined Doppler and two-dimensional imaging, the images were not clear enough for accurate measurement of the vessel diameter in a substantial portion of the examined subjects. Without the coronary artery diameter, we could only measure changes in coronary blood flow velocity, but not in coronary blood flow itself. Therefore, CFR assessment by transthoracic Doppler echocardiography is limited to measurement of coronary blood flow velocity. However, CFR measured using both parameters is closely correlated [16,17]. Another limitation is the feasibility of transthoracic coronary blood flow velocity measurement. Finally, the sample size was relatively small, and coronary angiography was performed in only 3 patients.

## Conclusions

Patch plasty without endarterectomy is an improvement over the conventional method, has results similar to conventional CABG, and is superior to endarterectomy; thus, it may be an alternative method for diffuse coronary artery disease. Transthoracic Doppler echocardiography is a feasible, noninvasive method that can be used in routine checkups of coronary bypassed patients.

### Consent statement

Written informed consent was obtained from the patient for publication of this research article and accompanying images. A copy of the written consent is available for review by the Editor-in-Chief of this journal.
